# Data Quality and Network Considerations for Mobile Contact Tracing and Health Monitoring

**DOI:** 10.3389/fdgth.2021.590194

**Published:** 2021-12-15

**Authors:** Riya Dave, Rashmi Gupta

**Affiliations:** Cognitive and Behavioural Neuroscience Laboratory, Department of Humanities and Social Sciences, Indian Institute of Technology Bombay, Mumbai, India

**Keywords:** digital health, mobile applications, COVID-19, contact tracing, AI

## Abstract

Machine Learning (ML) has been a useful tool for scientific advancement during the COVID-19 pandemic. Contact tracing apps are just one area reaping the benefits, as ML can use location and health data from these apps to forecast virus spread, predict “hotspots,” and identify vulnerable groups. However, to do so, it is first important to ensure that the dataset these apps yield is accurate, free of biases, and reliable, as any flaw can directly influence ML predictions. Given the lack of criteria to help ensure this, we present two requirements for those exploring using ML to follow. The requirements we presented work to uphold international data quality standards put forth for ML. We then identify where our requirements can be met, as countries have varying contact tracing apps and smartphone usages. Lastly, the advantages, limitations, and ethical considerations of our approach are discussed.

## Introduction

Contact tracing involves identifying infected individuals and those they were in contact with to halt virus transmission (1–3). Contact tracing traditionally involved paper-based methods and have been able to help combat outbreaks such as SARS in 2003 (4, 5), Ebola in Africa in 2014 (6), and smallpox (7). Given the prevalence of smartphones, countries worldwide have rushed to develop contact tracing apps to streamline and enhance the tracking process. These apps use GPS capabilities via Bluetooth on smartphones to collect location data on individuals (3, 8, 9). A risk assessment is conducted if an individual's smartphone is close to an infected individual's smartphone for a long enough time. The individual receives a notification on the next steps they should follow (e.g., self-quarantine or getting tested). Compared to paper-based methods, digital methods reduce the time involved in contacting a set of close contacts. Moreover, a systematic review found that contact tracing apps were less prone to data loss, opening paths for deeper health monitoring (10). Given the promising benefits, various countries have developed proximity-sensing applications to automatically trace contacts, notify users about potential exposures, and invite them to isolate (11).

### Prior Work in Digital Contact Tracing: Potential for Health Monitoring

Data collected from contact tracing apps can be evaluated in two primary ways. The first uses data analysis to perform a risk assessment that determines whether an individual should be notified of any exposure to the virus (3, 8, 9), thereby increasing the efficiency and accuracy of contact tracing methods. As case examples, such methods have been employed to control Ebola in Sierra Leone, tuberculosis in Botswana, and whooping cough (pertussis) in the USA. In addition, models that examined digital contact tracing have replicated disease outbreaks in schools and found that the digital system successfully identified participants' close contacts (10).

The second use of data collected from contact tracing apps involves deeper layers of health monitoring. Governments have taken the aggregated data collected from all users to observe and predict trends. By analyzing location and health data, this type of analysis can monitor the spread of the virus, predict “hotspots,” and forecast how resources should be distributed (5). From Ebola to the Zika virus to influenza, predictive analysis has enabled better resource allocation and public health planning (5, 6).

When the COVID-19 pandemic began, this effort was continued. For instance, models used location data in Wuhan to predict where the virus may arrive next in China. Other models strengthened their predictions by combining location data with other datasets, such as social media and credit card transactions, to successfully pinpoint vulnerable groups and predict hospital capacities (6, 7, 12).

### Data and Network Consideration

There are unquestionable benefits to applying data analysis for public health planning. However, it is well-known that any model is only as good as the data provided. Biases and inaccuracies, amongst other traits, can easily plague datasets and lead to faulty results. With predictive analysis, this is an ever-greater concern, as the data used for machine learning or model forecasting is not only an input but also a part of training the software itself (13). In other words, with predictive analysis, the overall system performance is assessed, at least primarily, on its dataset, leading any bias or inaccuracy to bring rise to large complications in how a country may distribute resources or declare “hotspots” (13, 14).

For this reason, international data quality standards specific to predictive analyses have been put forth. The standards guide predictive analysis by ensuring the dataset being used for predictive analysis is accurate, reliable, and comprehensive, amongst many other traits. Given the demand for predictive analysis, it is imperative that the datasets used to forecast health planning uphold such data measures. In this paper, we analyze how, in any part, health monitoring via contact tracing upholds data quality standards. We use the international standard of data quality measures to determine which measures are upheld when analyzing data from centralized servers (network topology consideration for data collection) for contact tracing apps. Even in decentralized servers all the decentralized data will be aggregated (data centralization in steps, to serve a large population with variable smartphone capabilities) to be consumed by ML in one way or the other. In addition to data quality standards, we also examine ethical considerations. Our objective is to break down and review data from centralized servers for health monitoring data quality and from an ethical standpoint.

However, apart from data and networks, many other factors might affect data collection and quality. The adoption of the contact tracing app is the most significant factor among them. The following are reasons why contact tracing app intervention failed in many countries, including the USA (15):

(a) Due to outdated laws about privacy, data collection, and intention to use data, contact tracing apps may not be deployed. (16).

(b) App developers may have faced difficulties devising an “acceptable to all solution” due to technological feasibility issues (e.g., type of network topology: centralized, decentralized, or hybrid for communication or to store the data; biased predictive algorithms; or the efficacy of communication channels: Wifi, Bluetooth, Ultrasound, etc.).

(c) People may not have wanted to download/use the application as intended due to distrust in the agency/government that was collecting the data, ethical concerns (misuse of the data, expiry of data), privacy concerns (e.g., surveillance), and over cybersecurity issues (e.g., hacking).

## Methods

### Evaluation of Network

Since ML models will be consuming the data generated by contact tracing apps, the centralized network topology is being considered. ML models both consume and generate a lot of data. With the emergency use of contact tracing apps during a pandemic and the current technology available, better prediction can only be achieved through centralized servers.

### Evaluation of Data Quality

International standards for data quality were surveyed to determine which quality dimensions to use. Articles put forth for quality dimensions specific to ML were analyzed. A list of potential quality dimensions was developed, and ultimately, the data quality model set forth by Rudraraju and Boyanapally (14) was predominantly used. The model was based on a widely used international data quality model (ISO/IEC 25012) and adapted to the specific needs of ML (14–17). Further details of this data quality model are delved into in the next section.

#### Criteria for Apps

To determine our criteria for ML application, research papers from PubMed, ScienceDirect, and NCBI were sought out using a combination of terms: contact-tracing, mobile applications, COVID-19, AI, ML, trend-analysis, servers, etc. Reference lists of papers and existing literature reviews were also referred to. Studies published in English on contact tracing apps or applying AI/ML to aggregated data were included (18). The criteria were developed after understanding how to best envelope the data quality dimensions put forth by Rudraraju and Boyanapally (14).

#### Global Adoption of Criteria

Before smartphones, other types of data (e.g., online news aggregators, expert-curated discussions, and official reports) were also used for epidemiology and predicting the spread of pandemics. For example, a website (HealthMap.org), operated by a team of researchers, epidemiologists, and software developers at Boston Children's Hospital, brings a unified and comprehensive view of the current global state of infectious diseases. This website uses a continuous automated process (e.g., monitors, organizes, integrates, filters, visualizes, and disseminates) and validated online information data sources (e.g., online news aggregators, expert-curated discussions, and official reports) to predict the current global state of infectious diseases (19).

To determine where our ML/AI application criteria can be met, we reviewed the smartphone penetration rate (percentage of the population actively using smartphones) and the contact tracing app's server type (centralized or decentralized) per country. ITU estimated that at the end of 2019, slightly more than 51 per cent of the global population, or 4 billion people, will be using the Internet; actual results are very close to the predicted one (20). Smartphone penetration rates were taken from Newzoo's Global Mobile Market Report, last updated in September of 2019 (21). The report lists the top 20 countries with the most active smartphone users along with corresponding smartphone penetration rates, which were taken for this review. An active smartphone user is qualified as an individual that uses the device at least once a month. The percent of smartphone users needed to use the contact tracing app to get to the 56% adoption rate (8, 30) was then calculated using the country's population of smartphone users (see **Table 2**). To determine server type, the main contact tracing app put forth by the government of that country was assessed through systematic searches, as some countries have second-party apps (22–27).

### Data Quality Dimensions for ML

As noted, to significantly forecast virus spread, the data used to train the ML model must be of quality, that is free of biases, inaccuracies, and inefficiencies, amongst many other traits, before it is analyzed (14). Noting the importance of quality data, international models have been put forth as standards for data scientists to follow. For this paper, we define quality data as data that upholds international and ML-based data standards.

The ISO (International Organization for Standardization) and the IEC (International Electrotechnical Commission) have put forth standardized dataset specifications in their Data Quality Model (ISO/IEC 25012) (17). This model has been widely deployed as a standardized data guideline and is the base of our quality dimension list (14). However, because this model was developed in the context of statistical studies where data is input instead of an architectural component, we also consider quality dimensions put forth for ML specifically (14, 28, 29).

While there are no standardized data quality models for ML, various authors recommended some models (13, 14). For our analysis, we decided to envelop the data quality dimensions for ML set forth by Rudraraju and Boyanapally (14). Their list integrates dimensions from the ISO/IEC 25012 model, interviews with a range of data scientists, and a thorough literature review of data quality attributes. Namely, the quality dimensions we aim to uphold are: Accuracy, Completeness, Credibility, Currentness, Efficiency, Traceability, Understandability, Availability, Reproducibility, Relevancy, Interpretability, Effectiveness, and Satisfaction. Definitions for each were taken from the work of Rudraraju and Boyanapally (14) and are listed in Table 1. For a deeper understanding of how these dimensions came to be, we direct the reader to the work of Rudraraju and Boyanapally (14).

**Table 1:** Data quality dimensions.

**Data quality dimension**	**Description**
Understandability	This attribute enables the users to interpret and express the information in appropriate languages and symbols for a specific context of use.
Fairness	The machine is trained with data with the ratio of all races (e.g., Black, white, etc.).
Currentness	This attribute identifies the information that is up to date.
Efficiency	Capability of providing suitable performance according to the number of resources used.
Availability	The degree to which the extracted data can be retrieved by authorized users for that context of use.
Relevance	To retrieve the data based on the requirement of the end-user or targeted customers.
Context Coverage	The level to which the system can be re-trained with the data that matches the end user's requirements.
Reproducibility	The degree to which the data can reproduce the same results and allow others to continue to train new machine learning systems.
Traceability	The extent to which the source of information, including owner and/or author of the information, and any changes made to the information can be verified.
Satisfaction	The extent to which the end-user is satisfied with the trained data.
Effectiveness	The capability to produce the desired output from the extracted data.
Completeness	The ability of data to represent every meaningful state of the represented real-world system.
Accuracy	Data is accurate when data values stored in the database correspond to real-world values or the extent to which data is correct, reliable, and certified.
Interpretability	To extract the data with the right language, units, and symbols with better understandability.
Credibility	The extent to which the information is reputable, objective, and trustable.
Size	Depending on the type of input data, the maximum amount of data that varies is the size of the data.

*This table shows the dimensions taken from Rudraraju and Boyanapally (14) that we used in this paper*.

## Analysis

### Ensure That the Contact Tracing App Can Achieve at Least a 56% User Adoption Rate

A group at the University of Oxford came up with an epidemiological simulation model to demonstrate the importance of contact tracing app intervention, indicating that delaying contact tracing by 1 day after the onset of symptoms could affect epidemic control and the resurgence of coronavirus. The model's assumptions and estimation of the key matrices (e.g., vaccination, lockdown, quarantine, other interventions, etc.) were derived from transmission dynamics analysis of early coronavirus outbreaks in China. The group measured successful outbreak control as a reduction in daily virus incidence, daily hospitalizations, number of people in or admitted to the hospital and ICU each day, daily deaths, number of people in quarantine each day, and number of tests required each day. This openly available model allows governments to compare and evaluate different contact tracing strategies alongside other real-time interventions (8). Any country may use this simulation model to derive/validate/estimate key matrices and use them for predictions.

Preliminary analysis of the UK National Health Service (NHS) Test and Trace programme at the Isle of Wight, by the same group, showed that contact tracing app intervention has a more significant impact on epidemic control. They concluded that there were significant decreases in incidence and R (basic reproduction number) (30). The group established the 56% adoption rate metric after investigating the effectiveness of contact tracing apps (8). This metric soon became the most cited adoption rate across literature, with the World Health Organization later stating that the adoption rate needed to be 60–70% (4, 9). The authors concluded that combining digital contact tracing with other interventions, such as community testing and continued shielding of vulnerable individuals, can help prevent coronavirus from rapidly re-emerging (8).

To further substantiate this metric, models examining contact tracing apps have shown that an adoption rate lower than 56% does not best represent a region's population, leading to virus resurgence and further lockdowns (8). As a case example, in a contact tracing app study conducted in the Isle of Man, while a 38% app adoption rate did improve aspects of the outbreak, authors noted that it did not effectively shut down virus spread. Although a 56% app adoption rate is far from complete usage by a population, it is clear that this metric brings about a sufficient and broad understanding of virus spread in the population that can be extrapolated from, mitigating the many possible data biases and inaccuracies that can arise.

A significant response to data biases and inaccuracies is necessary when working with data from contact tracing apps. Data riddled with biases can no longer be deemed fair. Further, it can no longer be considered a reliable, relevant, and complete representation of a population, which would generate long-term impacts on the data's effectiveness in stopping virus spread. For example, if ML was applied to understand how to best distribute resources to an incomplete dataset that did not represent the entire population, there would be an imperfect determination of “hotspots” (31). As a result, resource allocation of materials, such as testing kits and personal protective equipment, would be skewed (4, 32). Specifically, disproportionately more resources may be given to wealthier demographics that presumably have better access to smartphones than lower-income levels (33). It is thus imperative to have a majority adoption rate and possibly close to complete cooperation across ages and socioeconomic zones, as ML can not only make inaccurate predictions but also perpetuate stereotypes, deepening biases across gender, income, and race (30, 33). While an equitable distribution of smartphones may be logistically difficult to achieve, at a minimum, to work toward a credible dataset for ML to effectively act upon, we believe that at least 56% of a region's population must be using the contact tracing app. By achieving an app adoption rate of at least 56%, we believe that the following quality dimensions can be upheld: data completeness, relevancy, credibility, fairness, and effectiveness.

### Ensure the Contact Tracing App Has a Centralized Server to Store the Data

There are two dominating server types employed for contact tracing apps: centralized and decentralized systems. Each server has unique qualities and a differentiated ML approach. Before discussing why we believe a centralized system is better suited for upholding quality dimensions for data collected from contact-tracing apps, we will overview centralized and decentralized systems.

#### Centralized and Decentralized Servers

Amongst contact-tracing apps, there is global differentiation in what a centralized or decentralized server is (22). In a centralized server, data from the user is placed into a central source. In a decentralized system, location and contact data are stored locally on the user's smartphone (7). The process for either approach begins in the same way. When two smartphones come in contact with one another (Bluetooth range), a pseudonym code (anonymous identifier) is sent via Bluetooth to mark that interaction (4, 9). When individuals get tested for the virus, they can choose whether they want to upload the list of unique, anonymous identifiers to a common database in a decentralized model. This is the only information that the database receives, and with it, other phones can compare their unique identifiers with those of infected individuals to see if there is a possibility of exposure (34). However, in a centralized approach, the anonymous identifiers are uploaded along with proximity/interaction data (21, 34).

#### Deploying ML Application to Centralized Servers

As a result of the differentiated server types, ML is applied distinctly to each type. In centralized ML, the model is applied directly to the aggregated server, which holds the data. In decentralized ML, a model is sent to each smartphone. With the data on that phone, the model is then trained, and only model updates are sent to a central server, preventing any individual data from entering a single port. That updated model is then sent out to the next sequence of phones for training. In both, server communication—whether of data or model updates—is done through Bluetooth, Wi-Fi, or cellular networks. Further, in both, the trained model with the input data is then used to forecast virus spread (35).

#### Centralized Servers for Contact-Tracing Apps

Our analysis concludes that a centralized server is better suited to uphold data quality dimensions in contact tracing apps. Specifically, we believe that the following dimensions can be upheld: accuracy, efficiency, currentness, size, completeness, traceability, availability, interoperability, understandability, and integrability.

We begin with data completeness, currentness, and size. Contact tracing data must be up to date; any delay in gathering accurate health or location data directly impacts what we know about infected individuals in a community, leading to faulty ML predictions. ML would be applied to location and health data locally in a decentralized system—on each individual's phone. While this offers security benefits, as a centralized system would store the same location and health data in a single server for ML, there is a significant risk that location and health data from some users will not be accounted for (36–38). Suppose a user shuts off their phone, loses battery, or loses network connectivity during the round their phone has been selected for ML. In that case, the model will not take in that user's location and health data for its predictions until the next cycle.

Noting this concern, leading scientists have expressed that those who employ decentralized ML must be open to only a small subset of devices that may be active at each training round (36). While the same connectivity issues could exist with a centralized server (e.g., the phone could be powered down or out of network), a centralized system does not rely on a single point of data exchange for ML training. Updates are sent more continuously, allowing location and health data to be transferred once the phone returns to connectivity.

An additional issue for decentralized architectures is that delays would impact the currentness of the dataset, and any missing information would directly impact the size and completeness of the dataset. This concern is deepened through system heterogeneity–the term used to describe the fact that today's phones come from various manufacturers and that not all ML models can operate similarly on each phone, opening possibilities for data exclusion [for a review of system heterogeneity in health systems, refer to (37)]. We believe that a central port for data collection would help ensure that the location and health data used are up to date and inclusive of nearly all the available users when the ML model is applied.

Next, the dataset must be interoperable, understandable, and integratable. This is especially critical in forecasting virus spread; so as to strengthen predictions of clusters of cases or vulnerable groups, many ML models have combined contact tracing data with other datasets, such as credit card transactions or social media. While a large, aggregated server is more susceptible to complications from a crash, creating an integrated port for data collection makes it easier to combine non-homogeneous data. To elaborate on this point, not all local phones may have the capability to combine outside data readily and feasibly, and doing so would put in question data privacy advantages in a decentralized system. A central port allows data to be cleaned then analyzed together, ridding the architectural restraints of configuring a system locally on each phone.

A central port also helps ensure that the data is traceable and readily available. When contact tracing, it is integral to have a method to trace back to the source of an infection or outbreak. Doing so makes it possible to build upon what contact tracing apps do with other methods—contacting family members in that household or determining new biomarkers. Through a single aggregated server, it is possible to have a way to trace back data to the point of an issue or a specific user anonymously. This directly contrasts to localized information storage, a strategy that promotes privacy but makes it difficult to locate a datapoint anonymously (36–38).

Lastly, variations in data points between individual phones and communication bottlenecks have led us to deem centralized servers as more efficient for contact tracing apps. With efficiency and currentness, the ML model can be quickly updated to build the best predictions. In decentralized ML, rounds of updates must be sent back to the central model, leading communication efficiency to be a known bottleneck. In addition, variations in data points collected tamper streamlining the ML process as a whole, as the data may not be homogeneously sent or “understandable.” Data satisfaction is essential for the app's end-user—the general people—and ensuring that data is communicated well is imperative.

Due to challenges with expensive communication, system heterogeneity, and interoperability, amongst other areas, we believe that a centralized server is better suited for ML application to data collected from contact tracing apps. While a decentralized system offers advantages, mainly when privacy and data currentness are not of central concern, our focus is on maintaining international standards for applying ML to contact tracing datasets. A centralized server will help us do exactly that, consequently better ensuring that a contact tracing model can continuously produce accurate, reliable, and consistent predictions to combat virus spread.

## Where Is It Possible to Meet the Requirements?

Given the large variability in how countries are developing their apps, it is important to note that not all countries will be using a centralized server. In addition, given the large variability in smartphone penetration rates, not all countries will achieve an app adoption rate of 56%. To reach an adoption rate of 56%, at least 56% of a country's population must have access to a smartphone and use the app through a forced mandate or voluntarily. The data in Table 2 highlights the global differentiation in server type as well as smartphone usage. Of the countries listed, we see that France, South Korea, Russia, and China meet both the requirements (highlighted in green, Table 2). All countries above the red line have a smartphone penetration rate of at least 56%. The United States is marked with an asterisk because while certain states have begun to deploy apps, there is no national consensus on whether the apps should be implemented (24).

**Table 2:** Applying ML to data from Contact-Tracing Apps.

**Country**	**Smartphone penetration rate (%)**	**Decentralized or centralized?**	**Meets our standards to apply AI?**	**Percent of smartphone users needed to meet threshold of 56% adoption rate (%)**
United Kingdom	82.9	Decentralized	No	67.6
Germany	79.9	Decentralized	No	70.0
United States	79.1	Decentralized*	No	70.8
France	77.5	Centralized	Yes	72.3
Spain	74.3	Decentralized	No	75.3
South Korea	70.4	Centralized	Yes	79.6
Russia	66.3	Centralized	Yes	84.5
Italy	60.8	Decentralized	No	92.1
China	59.9	Centralized	Yes	93.4
Japan	57.2	Decentralized	No	97.9
Iran	54.8	Centralized	No	102.1
Turkey	54.0	Centralized	No	103.8
Mexico	49.5	————–**	No	112.9
Brazil	45.6	Decentralized	No	122.7
Vietnam	44.9	Decentralized	No	124.8
Philippines	33.6	Decentralized	No	166.8
Indonesia	31.1	Centralized	No	179.9
India	36.7	Centralized	No	152.6
Bangladesh	18.5	Centralized	No	303.7
Pakistan	15.9	Centralized	No	352.5

*The smartphone penetration rate per country and its server type to store contact tracing data. Countries listed above the red line have a smartphone penetration rate of at least 56%. If a country has a centralized server, AI can be feasibly applied to the data (denoted by “yes” and green box). The percentage of smartphone users required to achieve a 56% adoption rate is also listed*.

* *In the United States, it should be noted that while certain states have begun to design official contract tracing apps, there is not a national consensus*.

** *Information on Mexico could not be retrieved*.

## Limitations

### Limitations of the Geographical Findings

The Newzoo report only included 20 countries: those with the most active smartphone users. Thus, countries with smaller populations and high smartphone penetration rates were excluded.

First, it is essential to consider how those countries not listed are implementing contact tracing apps to gain a complete global understanding. For example, in Australia, the smartphone penetration rate is estimated to be 80%, and leaders have leaned toward a centralized server for their contact tracing app (39, 40). Secondly, while widely deployed, the use of contact tracing is still a developing effort, as decisions by leaders are amenable to current updates on data privacy and app efficacy. Just as the United Kingdom shifted from a centralized to decentralized approach, Germany and Austria ultimately decided to go with a decentralized approach (22). Lastly, it has been shown that COVID-19 tracking systems do not capture data on immigrants and other marginalized populations (33). Other groups without smartphones, such as the elderly and children under the age of 10, are also not accounted for in any analysis by AI on contact tracing data (8).

### Limitations of the Approach

Despite the many advantages, we acknowledge that using a centralized server and ensuring a high user adoption rate is no panacea to ensuring quality data for virus forecasting. The accuracy of the dataset itself is dependent on a myriad of other factors, such as widespread testing, guaranteed app adoption rate, and the app's efficacy (4, 9). In cases where there is not widespread and efficient testing, individuals will struggle to get tested despite the app's recommendations, making it difficult to identify infected individuals and break the chain of transmission (4, 9, 34). In addition, even if there is a smartphone penetration rate of at least 56% in the country, the app's efficacy will only go as far as the number of individuals that agree to download it.

Lastly, despite the proposed benefits of the app itself, there is uncertainty around its true efficacy. The concern is two-fold: (1) There is little, if any, risk assessment or validation done on these apps before launch and (2) There are design limitations (4, 9, 31). For the first point, due to the urgency of the pandemic, countries have rolled out contact tracing apps without a proper assessment of the accuracy and success of the product (4). Will the app accurately track individuals, and should ML be applied if it does not? The second concern surrounds the design limitations of smartphones, and thus, the app itself. GPS/Bluetooth capabilities cannot account for situations in which individuals hold the same geolocation but are spatially distanced (31). For example, two individuals could be separated by a wall or on different floors of a building. Further consideration must be given to the management of false positives, as this would directly impact quality standards, such as data completeness and credibility.

## Ethical Considerations

In applying AI to data collected from a centralized server, ethical considerations must be addressed (23, 25–27, 39, 41). As mentioned, a decentralized server offers greater privacy because the data is processed locally: on the user's phone. Therefore, a centralized server must encrypt the data and have high-security protocols due to high susceptibility to data breaches (4, 41). Without security protocols, an individual's right to privacy is violated. As case examples, South Korea and Qatar have recently scrutinized security loopholes found in their apps and the implications on individuals (42). While it has also been shown that decentralized models can also be susceptible to similar data breaches, more research needs to be conducted to gain insight into this field. Furthermore, while the app data is typically anonymized, it has been shown that machine learning can re-identify data, leading to ethical concerns over privacy rights (42, 43).

Next, there is no clear definition of when data should be deleted from the central server. The World Health Organization suggests that data be deleted after the pandemic has ended locally (9). Given the large uncertainty of when that could be, questions surrounding public surveillance and the duration and ease of that surveillance arise (4). How long could governments track individuals, and can individuals ever ask that their data be taken off the server? Is it justifiable to continue to use ML to analyze the data even after the pandemic subsides? What if there is mission creep–analyzing the data outside the defined scope?

Lastly, to achieve greater adoption, countries such as Qatar have decided to mandate the use of the contact tracing app (44). Ethical analyses are necessary to understand whether it is justified to mandate the use of an app despite violations of individual rights. While a mandate would ensure that a majority uses the app, certain individual rights, such as privacy and liberty, would be infringed upon in the process (26).

## Conclusion

We have presented a proposed method for using ML to analyze data from contact tracing apps consistent with data quality standards. In addition, we have identified the countries in which our methods are most feasible, later discussing ethical considerations, advantages, and limitations of this approach. As the pandemic rages on, it is ever more critical that ML models analyze quality contact tracing data. We hope to shed light on the need for a methodological approach, inspiring further research into this field.

## Data Availability Statement

Publicly available datasets were analyzed in this study. This data can be found here: https://resources.newzoo.com/hubfs/Reports/2019_Free_Global_Mobile_Market_Report.pdf?utm_campaign=Mobile%20Report%20Launch%202019&utm_medium=email&_hsmi=76926953&_hsenc=p2ANqtz-9srHwQONqYM2EChvObOaegqly7JvX3KNYKSHWJKFR2j-iOvj6TW4uDROWXR-OJw8oOw_7j9KHakgEYu_NATW8-Q3TW0Q&utm_content=76926953&utm_source=hs_automation.

## Author Contributions

All authors have written the paper.

## Conflict of Interest

The authors declare that the research was conducted in the absence of any commercial or financial relationships that could be construed as a potential conflict of interest.

## Publisher's Note

All claims expressed in this article are solely those of the authors and do not necessarily represent those of their affiliated organizations, or those of the publisher, the editors and the reviewers. Any product that may be evaluated in this article, or claim that may be made by its manufacturer, is not guaranteed or endorsed by the publisher.
